# Does Beef Protein Supplementation Improve Body Composition and Exercise Performance? A Systematic Review and Meta-Analysis of Randomized Controlled Trials

**DOI:** 10.3390/nu11061429

**Published:** 2019-06-25

**Authors:** Pedro L. Valenzuela, Fernando Mata, Javier S. Morales, Adrián Castillo-García, Alejandro Lucia

**Affiliations:** 1 Department of Systems Biology, University of Alcalá, 28805 Madrid, Spain; pedrol.valenzuela@edu.uah.es; 2 NutriScience, Nutrition & Health Sciences, 14002 Córdoba, Spain; fernando.mata@nutriscience.pt; 3 Faculty of Sport Sciences, Universidad Europea de Madrid and Research Institute ‘i+12’ (Hospital 12 de Octubre), 28670 Madrid, Spain; javiersalvador.morales@universidadeuropea.es; 4 Fissac-Physiology, health and physical activity, 28015 Madrid, Spain; castillogarcia.adrian@gmail.com

**Keywords:** resistance training, exercise, nutrition, muscle mass, hypertrophy

## Abstract

Protein supplementation might improve body composition and exercise performance. Supplements containing whey protein (WP) have received the most attention, but other protein sources such as beef protein (BP) are gaining popularity. We conducted a systematic review and meta-analysis of randomized controlled trials that compared the effects of exercise training combined with BP, WP or no protein supplementation (NP), on body composition or exercise performance. Secondary endpoints included intervention effects on total protein intake and hematological parameters. Seven studies (*n* = 270 participants) were included. No differences were found between BP and WP for total protein intake (standardized mean difference (SMD) = 0.04, *p* = 0.892), lean body mass (LBM) (SMD = −0.01, *p* = 0.970) or fat mass (SMD = 0.07, *p* = 0.760). BP significantly increased total daily protein intake (SMD = 0.68, *p* < 0.001), LBM (SMD = 0.34, *p* = 0.049) and lower-limb muscle strength (SMD = 0.40, *p* = 0.014) compared to NP, but no significant differences were found between both conditions for fat mass (SMD = 0.15, *p* = 0.256), upper-limb muscle strength (SMD = 0.16, *p* = 0.536) or total iron intake (SMD = 0.29, *p* = 0.089). In summary, BP provides similar effects to WP on protein intake and body composition and, compared to NP, might be an effective intervention to increase total daily protein intake, LBM and lower-limb muscle strength.

## 1. Introduction

Protein supplementation might increase muscle anabolism and, if combined with exercise training, could also improve physical performance [1,2,3]. The anabolic response to protein intake can be affected not only by factors such as individual nutritional state, digestive/absorption capacity, or sensitivity of muscle anabolic pathways, but also by the source of protein [4]. In this regard, numerous protein sources have been investigated for their effect on muscle anabolism, including milk [5], eggs [6], soy [7], rice [8] or bovine colostrum [9]. Although supplements containing whey protein (WP) have received the most attention due to their high content in leucine and potential anabolic effect [10,11], beef protein (BP) has gained popularity in recent years [12].

Acute ingestion of BP can increase muscle protein synthesis in both young and older individuals [13,14,15], and these benefits seem to be maximized when combined with exercise training (particularly resistance training) [16]. There is thus biological rationale to support BP supplementation as a potentially effective strategy for improving lean body mass (LBM) and physical performance (notably, muscle strength). However, the evidence on the effectiveness of BP supplementation for increasing muscle mass or performance is mixed, with some studies reporting benefits compared to no protein supplementation [17,18] but others finding no such benefits [19,20]. Moreover, the effectiveness of BP for improving body composition or performance compared to WP remains unclear [18,19,21,22].

On the other hand, owing to its high content in heme iron, BP supplementation could also increase total iron intake and thus theoretically benefit hematological parameters, and indeed some benefits have been reported [20,22]. However, to our knowledge, there is not yet meta-analytical evidence on the potential benefits of BP supplementation.

Given the purported benefits of BP supplementation on body composition and performance, and the lack of consensus on its actual effectiveness, the main aim of this systematic review and meta-analysis was to compare the effects of BP, WP or no protein supplementation (NP) combined with exercise training on body composition and exercise performance. Changes in nutritional intake and hematological parameters were also analyzed as secondary endpoints.

## 2. Materials and Methods 

The conduct and reporting of the current systematic review and meta-analysis conform to the Preferred Reporting Items for Systematic Reviews and Meta-Analyses (PRISMA) [23].

### 2.1. Systematic Search

Relevant articles were identified by title and abstract in the electronic databases MEDLINE, Web of Science and SportDiscus (since inception to 5 May 2019) using the following search strategy: (beef OR meat) AND (supplement*) AND (athlet* OR train* OR “physical activity” OR exercise). The electronic search was supplemented by a manual review of reference lists from relevant publications and reviews to find additional publications on the subject. Gray literature (abstracts, conference proceedings or editorials) and reviews were excluded. 

### 2.2. Study Selection and Data Extraction

Studies were eligible for inclusion if they met each of the following criteria: (a) analyzed the effect of BP supplementation on at least one endpoint related to body composition or exercise performance; (b) duration of the exercise training + supplementation intervention ≥4 weeks; (c) used a randomized controlled trial design that included one group performing exercise training in combination with BP supplementation and 1+ groups performing the same exercise program as those receiving BP, but with WP or NP. Case studies were excluded, as well as those in which BP was combined with WP or other protein/‘anabolic’ supplements (e.g., creatine). 

Two reviewers (P.L.V. and F.M.) independently extracted the following data from each study: number of participants within each group, participants’ characteristics, exercise and nutritional intervention’s characteristics, endpoints, measurement methods and results. Disagreements were resolved through discussion with a third reviewer (J.S.M.).

Endpoints data were registered as mean and standard deviation (SD). When the standard error of the mean (SEM) but not the SD was available, the latter was calculated from the square root of the sample size multiplied by the SEM. If data were provided as a figure, we used a specific software (WebPlotDigitalizer 4.2, San Francisco, CA, USA) for data extraction. We contacted the authors when necessary to request additional information. In this regard, the authors of two studies [17,19] provided us with the required specific data upon request. Fat-free mass was taken as a surrogate of LBM if the latter was not reported.

### 2.3. Quality Assessment 

Study quality was evaluated using the ‘risk-of-bias’ assessment tool following the recommendations by the Cochrane Handbook for Systematic Reviews of Interventions [24]. Two authors (A.C.G. and J.S.M.) analyzed the following criteria: random sequence generation and allocation concealment (selection bias), blinding of participants and research staff to group allocation (performance bias), blinding of outcome assessor (detection bias), incomplete outcome data (attrition bias), and selective reporting (reporting bias). Quality assessment by both reviewers was compared and disagreements in scores were resolved through discussion with a third reviewer (P.L.V.). 

### 2.4. Statistical Analysis

We performed a meta-analysis when a minimum of three studies compared the effects (pre- and post-intervention data) of BP vs. WP or NP for a given endpoint. The pooled standardized mean difference (SMD) between interventions (post- *minus* pre-intervention data) and 95% confidence interval (CI) were computed using a random effects model. A conservative Pearson’ *r* value of 0.7 was used for the computation of the within-group SD [25], and a sensitivity analysis (*r* = 0.2, 0.5 or 0.9) was then performed when a significant result was found. Begg’s test was used to determine the presence of publication bias. We considered that the analysis of an outcome was at risk of bias when Begg’s test was significant (i.e., *p* < 0.05). The *Q* and *I*^2^ statistics were used to assess heterogeneity between studies. *I*^2^ values of 25, 50, and 75% were considered reflective of low, moderate, and large heterogeneity, respectively.

All statistical analyses were performed using the statistical software package Comprehensive Meta-analyis 2.0 (Biostat; Englewood, NJ, USA) setting the level of significance at 0.05.

## 3. Results

### 3.1. Included Studies

**Figure 1:**
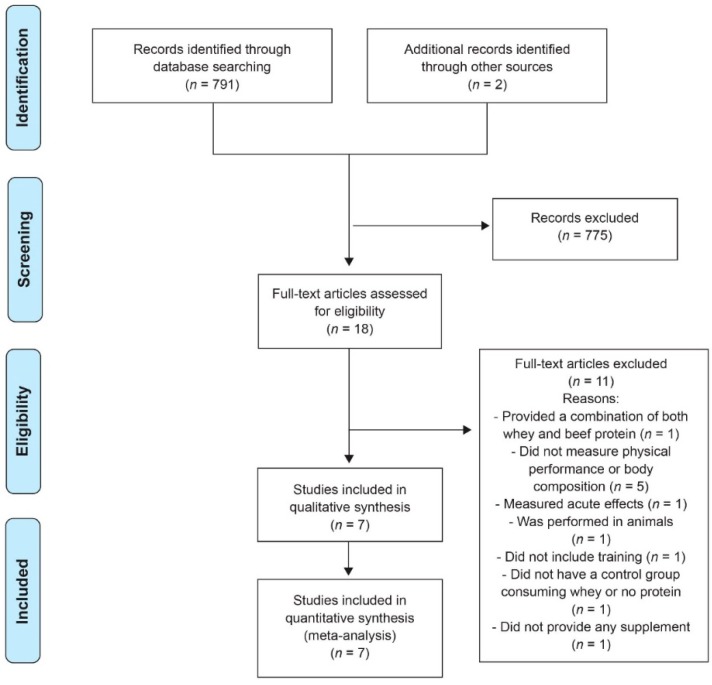
Flow chart of literature search.

From the retrieved articles, seven (including 270 participants in total) [17,18,19,20,21,22,26] met all inclusion criteria and were included in the systematic review (Figure 1, Table 1).

### 3.2. Quality Assessment and Publication Bias

**Figure 2:**
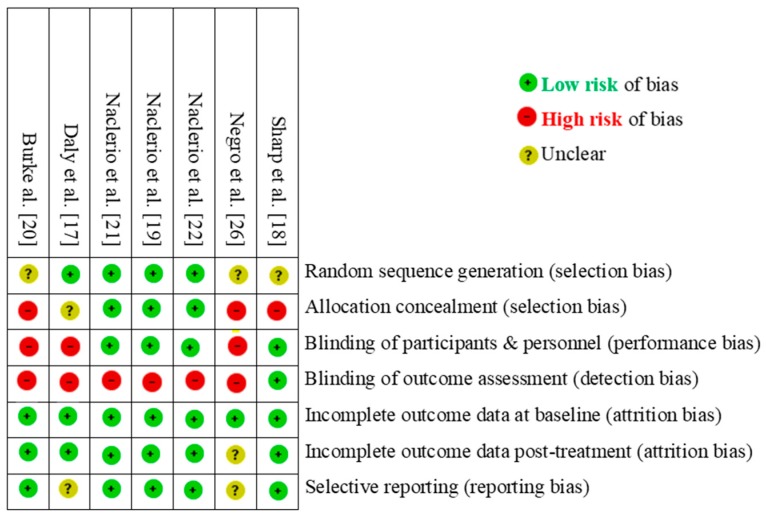
Quality assessment of the included studies.

The risk of bias in the included studies was overall low (Figure 2). Three of seven studies [18,20,26] failed to perform/report an appropriate random sequence generation or allocation concealment. Three studies (where BP was provided as lean beef) were at a high risk of performance bias because participants were not blinded to group allocation [17,20,26]. 

### 3.3. Participants and Intervention Characteristics

Five studies [18,19,20,21,26] analyzed young male and female active/trained subjects whose age ranged between ~18 and 30 years, one [22] analyzed master male athletes (35 to 60 years), and another one [17] assessed women aged 60 to 90 years.

The duration of the interventions ranged from 8 to 16 weeks. BP was supplied as isolated BP (powder form) in four studies [18,19,21,22] or as a lean beef supplement in a remainder of studies [17,20,26]. The amount of protein was specified in all but one study [20], and ranged from 16.4 to 46 g/day. BP was compared with NP in all studies, and with WP in four of them [18,19,21,22]. The nutritional intervention was combined with a resistance (‘strength’) exercise training program (2 to 3 sessions/ week) in five studies [17,18,19,21,26] or with aerobic exercise training (four to six sessions/week) in one study [22]. One study [20] only reported that participants were cross-country runners who maintained their typical usual exercise regime during the intervention.

### 3.4. Body Composition

**Figure 3:**
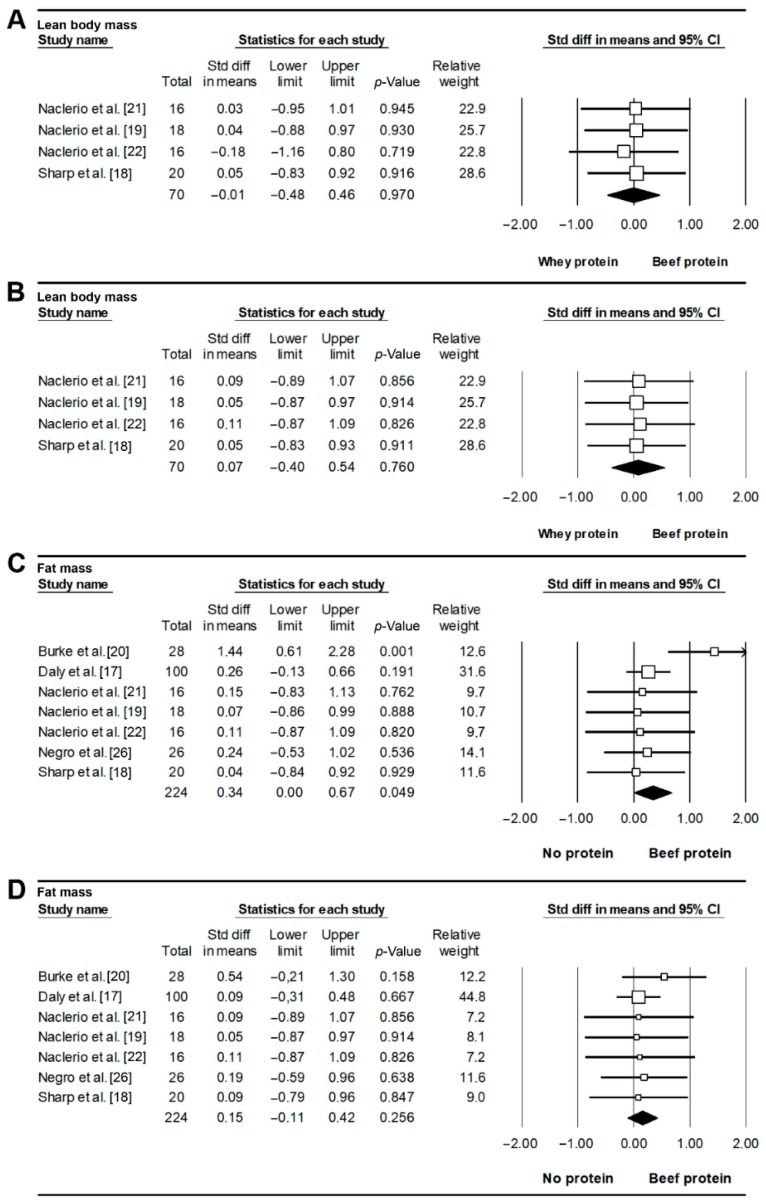
Effects on lean body (**A**) and fat mass (**B**) of beef vs. whey protein supplementation and effects on lean body (panel **C**) and fat mass (panel **D**) of beef vs. no protein supplementation. Each forest plot displays the pooled standardized difference in means and 95% confidence interval.

Four studies [18,19,21,22] compared the effects of BP and WP on body composition, with none reporting significant differences. Thus, the meta-analysis showed no differences between both conditions for LBM (SMD = −0.01, *p* = 0.970, Figure 3A), with no signs of heterogeneity (*I*^2^ = 0%, *Q* = 0.152) or publication bias (*p* = 0.202). Similarly, no differences were found between BP and WP for fat mass (SMD = 0.07, *p* = 0.760, Figure 3B), with no signs of heterogeneity (*I*^2^=0%, *Q* = 0.012) or publication bias (*p* = 0.054).

All included studies compared the effects of BP vs. NP on body composition. The meta-analysis showed a beneficial effect of BP over NP for LBM (SMD = 0.34, *p* = 0.049, Figure 3C), with no signs of heterogeneity (*I*^2^ = 4.326%, *Q* = 6.271) or publication bias (*p* = 0.500). This effect remained significant for *r* = 0.9 (SMD = 0.52, *p* = 0.032) or almost significant for 0.5 (SMD = 0.26, *p* = 0.051), but differences did not reach statistical significance for *r*=0.2 (SMD = 0.21, *p* = 0.112). No differences were found between BP and NP for fat mass (SMD = 0.15, *p* = 0.256, Figure 3D), with no signs of heterogeneity (*I*^2^ = 0%, *Q* = 1.238) or publication bias (*p* = 0.500).

Three studies [19,21,22] analyzed changes in muscle thickness using ultrasonography, but we could not meta-analyze their results because these studies analyzed different muscles. One study [19] found no benefits on muscle thickness with BP vs. WP or NP. However, the other two reported an improvement in the thickness of the *biceps brachialis* [21] or *vastus medialis* muscle [22] with BP compared to WP or NP. One study [22] also analyzed changes in thigh and arm circumference and found larger benefits with BP vs. WP or NP. Finally, one study [17] that analyzed thigh muscle cross-sectional area as well bone mineral density (femoral neck, total hip and lumbar spine) using dual-energy X-ray absorptiometry found no differences between BP and NP.

### 3.5. Exercise Performance

**Figure 4:**
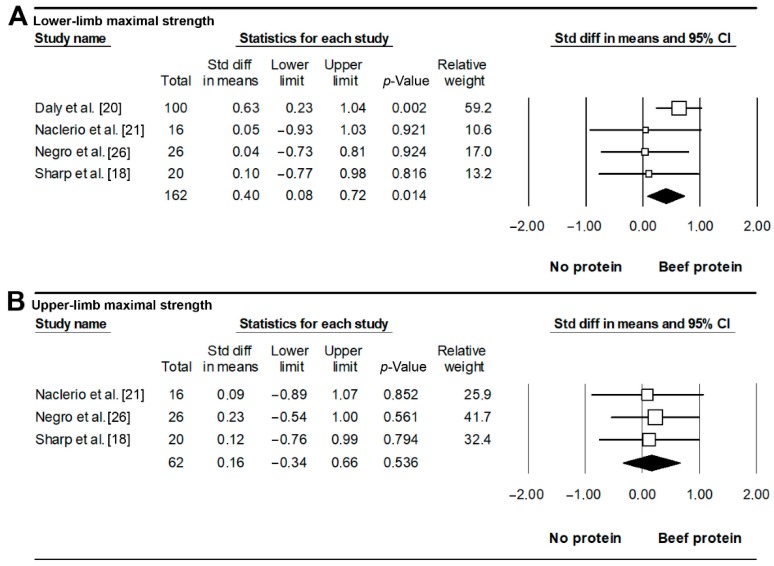
Effects on lower- (**A**) and upper-limb maximal strength (**B**) of beef vs. no protein supplementation. Each forest plot displays the pooled standardized difference in means and 95% confidence interval.

All retrieved studies analyzed the effects of BP on 1+ markers of exercise performance. Four studies [17,18,21,26] analyzed the effects of BP vs. NP on lower-limb maximal strength (deadlift, leg press, knee extension). Of these, three [18,21,26] found similar increases in strength with both conditions, and one study [17] found an 18% greater increase in leg extension strength with BP than with NP. The meta-analysis showed a beneficial effect of BP over NP (SMD = 0.40, *p* = 0.014, Figure 4A), with no signs of heterogeneity (*I*^2^ = 0%, *Q* = 2.954) or publication bias (*p* = 0.367). This effect remained significant for *r*=0.5 (SMD = 0.33, *p* = 0.040), but not for *r* = 0.2 (SMD = 0.26, *p* = 0.098) or 0.9 (SMD = 0.42, *p* = 0.150). Three studies [18,21,26] that assessed changes in upper-limb maximal muscle strength (bench press, 1-repetition maximum [1RM]) found no differences between BP and NP, and the meta-analysis showed no differences between both conditions for this outcome (SMD = 0.16, *p* = 0.536, *I*^2^ = 0%, *Q* = 0.058, Begg’s *p*-value = 0.148, Figure 4B). 

Two studies [18,21] also compared the gains in 1RM with BP over WP and neither found additional benefits. Finally, no benefits were reported with BP vs. NP for maximal oxygen uptake [20,22], anaerobic peak power [18] or total weight lifted during a resistance training session [19].

### 3.6. Nutritional Intake

**Figure 5:**
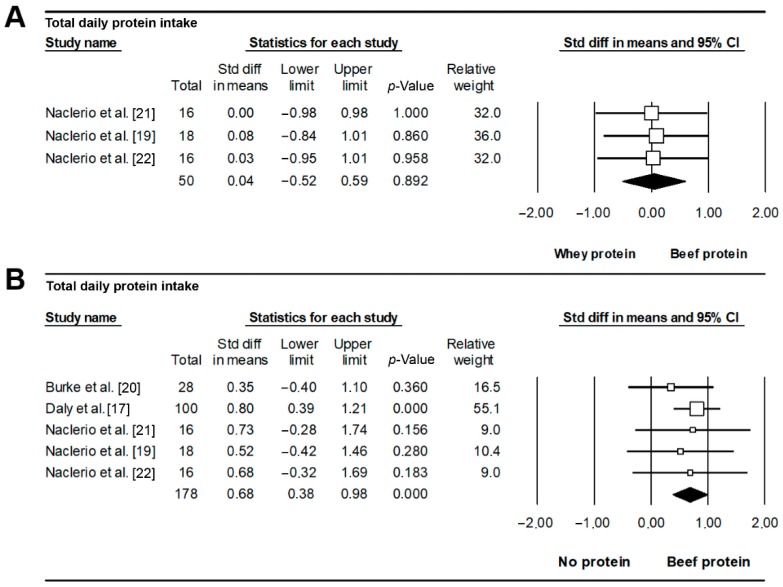
Effects on total daily protein intake of beef vs. whey (**A**) or no protein supplementation (**B**). Each forest plot displays the pooled standardized difference in means and 95% confidence interval.

Except for one study [26], where the baseline protein intake of the participants was described as ~1.0 g/kg/day, the mean total protein intake (in g/kg/day) during the intervention was consistently reported and ranged from 1.3 to 2.2 g/kg/day (BP), 1.7 to 2.2 g/kg/day (WP), and 1.1 to 2.0 g/kg/day (NP). The total protein intake was higher in BP than NP (SMD = 0.68, *p* < 0.001, Figure 5A; *I*^2^ = 0%, *Q* = 1.226, Begg’s *p* = 0.403). This effect remained for *r* = 0.2 (SMD = 0.42, *p* = 0.006), 0.5 (SMD = 0.53, *p* = 0.001) or 0.9 (SMD = 1.17, *p* < 0.001). By contrast, no differences in protein intake were found for BP vs. WP (SMD = 0.04, *p* = 0.892, Figure 5B), with no heterogeneity between studies (*I*^2^ = 0%, *Q* = 0.016) and no signs of publication bias (*p* = 0.500).

Five studies [17,19,20,21,22] analyzed changes in fat intake with BP vs. NP, with no significant differences between conditions (SMD = −0.11, *p* = 0.587) and with no heterogeneity between studies (*I*^2^ = 3.8%, *Q* = 4.158) and no publication bias (*p* = 0.110). Three of these studies [19,21,22] also analyzed the changes in fat intake with BP vs. WP, and the meta-analysis showed a higher fat intake with the former (SMD = 0.71, *p* = 0.015), with no heterogeneity (*I*^2^ = 0%, *Q* = 0.195) or bias between studies (*p* = 0.148). This effect remained significant for *r* = 0.9 (SMD = 1.22, *p* < 0.001), but not for 0.5 (SMD = 0.55, *p* = 0.056) or 0.2 (SMD = 0.44, *p* = 0.128).

Three studies [17,20,22] analyzed changes in iron intake with BP vs. NP. The meta-analysis showed a non-significant trend towards a higher iron intake with BP (SMD = 0.29, *p* = 0.089), which was significant for *r* = 0.9 (SMD = 0.46, *p* = 0.006), but not for *r* = 0.5 (SMD = 0.22, *p* = 0.180) or 0.2 (SMD = 0.18, *p* = 0.285).

### 3.7. Hematological Parameters

Two studies [20,22] analyzed the effects of BP vs. NP on hematological parameters, with one study reporting an increased ferritin concentration [22] and the other study finding an increased hematocrit (although significance was only reached for the female participants) [20]. 

Three studies [17,18,19] analyzed the effects of BP vs. NP on lipid profile, and two of them [18,19] also compared BP and WP. None of them found differences between interventions for any of the analyzed variables (i.e., total cholesterol, LDL and HDL-cholesterol, or triglycerides). However, no meta-analysis could be performed because the mean and SD values for baseline and post-intervention were not available for all the studies. 

## 4. Discussion

Protein supplementation might be a potentially beneficial strategy for maximizing exercise training-related gains in body composition and performance [2], with WP receiving the most attention to date. This systematic review and meta-analysis showed that, when combined with exercise training, BP supplementation provides benefits on protein intake and LBM that are similar to those elicited by WP. Our results also show that BP supplementation might be an effective means of increasing total daily protein intake compared to NP, and suggest it might be also useful for improving LBM and lower-limb muscle strength, although these results should be corroborated in further studies. 

The popularity of WP compared to other protein sources is mainly based on a higher digestibility of the former together with a greater content of essential amino acids such as leucine [27]. Indeed, WP has proven to stimulate muscle protein synthesis to a greater extent than other popular protein sources such as casein or soy [28,29]. The ingestion of BP has also demonstrated promising anabolic effects, with the ingestion of 30 g (equivalent to 113 g of lean beef) increasing muscle protein synthesis by ~50% compared with fasting conditions [13,14,15]. Moreover, the combination of BP and exercise training might provide additional benefits, as the increase in muscle protein synthesis observed after BP intake followed by a resistance training session is higher than that observed with the former alone [15,16]. However, WP has been reported to have a higher digestibility (as reflected by a higher Protein Digestibility Corrected Amino Acid Score, 1.00 vs. 0.92) as well as a greater content of essential amino acids than BP (52% vs. 44% of total protein, respectively), including more leucine (13.6% vs. 8.8%) and lysine (10.6% vs. 8.9%) and a similar methionine content (2.5% vs. 2.3%) [27]. Of note, in human muscle, essential amino acids make up to 45% of the total protein content, and 9.4%, 8.7% and 2.2% of the protein comes from leucine, lysine and methionine, respectively [27]. Burd et al. [30] compared the mean muscle protein synthesis response to the ingestion of 30 g of protein coming from skimmed milk or beef after a resistance training session. Skimmed milk (from which WP is obtained) was more rapidly digested and absorbed than beef, thereby resulting in a greater leucine availability, and a higher stimulation of muscle protein synthesis in the early phase after exercise (0–2 h), but not for the whole recovery period (0–5 h post-exercise) [30]. Thus, these results suggest that milk/WP might be more effective than BP for the stimulation of muscle anabolism during the early postprandial stage, but that no overall differences are found between these protein sources. In line with these findings, our results support similar mid/long-term (≥4 weeks) benefits of WP or BP supplementation on LBM.

On the other hand, the present study shows that BP might be effective compared to NP for increasing LBM and lower-limb muscle strength, although the magnitude of the benefit was small (SMD <0.50 in both cases) and this result should be confirmed in further studies as sensitivity analyses raised concern about statistical significance. Furthermore, no consistent benefits were observed on physical performance, as reflected by the lack of differences for upper-limb muscle strength. Given the abovementioned anabolic potential of BP, which also resulted in a higher total daily protein intake than NP, we expected greater benefits of BP on LBM and physical performance compared to NP. One of the factors that might explain the small benefits obtained in these endpoints is that participants already consumed an ‘optimal’ quantity of protein in their diet (1.1 to 2.0 g/kg/day for the NP group, above the current international recommendations for the general adult population of 0.8 g/kg/day [31]). Of note, a protein intake higher than 0.8 g/kg/day is recommended to promote skeletal muscle anabolism in physically active individuals (i.e., 1.0, 1.3, and 1.6 g/kg/day for individuals with minimal, moderate, and intense physical activity levels, respectively; [32]) as well as in older people (>1.2 g/kg/day, [33]) such as those included in the study of Daly et al. [17]. However, a recent meta-analysis found no additional benefits on LBM when protein intake was greater than 1.6 g/kg/day [2]. Thus, the daily protein intake of the participants in the studies analyzed here could be considered within the ‘optimal’ range even without protein supplementation. Greater benefits might perhaps have been observed in individuals with a baseline protein intake below the recommended levels.

Another purported benefit of supplementation with BP is the potential increase in iron intake, which might theoretically improve hematological parameters. In this regard, our results show a non-significant trend towards an increased iron intake with BP compared to NP. Two studies [20,22] analyzed the specific changes in heme iron intake and both found increased values with BP—although only in the female participants in one study [20]—compared to WP or NP. In line with these results, although we could not perform a meta-analysis of hematological parameters, preliminary evidence suggests that BP could provide benefits at hematological level, such as increased hematocrit levels in collegiate distance runners [20] or enhanced iron (ferritin) deposits in master-age triathletes [22]. Future research should confirm if BP might serve as an effective intervention to counteract hematological conditions such as anemia in individuals with deficient iron intake.

An interesting finding was that, when compared to WP, BP supplementation seemed to increase the total daily intake of fat. However, in sensitivity analysis, this effect did not reach statistical significance and no differences in total daily fat intake were observed between BP and NP. Thus, there is not yet clear evidence to support that BP *per se* significantly increases fat intake. Moreover, no differences have been reported in lipid profile (e.g., cholesterol levels) or in fat mass with BP compared to WP or NP. However, more research is needed in the field.

Some limitations must be noted, notably the small number of studies included, the small sample size of most studies or the lack of participants’ blinding to group allocation in some of them. The included studies provided BP in different forms (powder or lean beef), which might have affected digestibility/absorption rates and potentially, anabolic responses [34]. However, although minced beef is more rapidly digested/absorbed than beef steak resulting in a greater amino acid availability, no differences have been reported in post-prandial muscle protein synthesis [35]. Future research should confirm if providing BP in isolate form (powder) results in a greater anabolic response than lean beef. The type of exercise training intervention did also differ between studies, with some including resistance training and others using endurance training. In this regard, resistance training is more effective for promoting muscle protein synthesis and muscle mass accretion than endurance exercise [36], and this might have acted as a confounding variable in our findings. However, protein supplementation can also provide benefits in individuals performing endurance exercise training, notably by avoiding muscle catabolism—especially during periods of negative energy intake—and through the enhancement of recovery and the promotion of exercise-induced adaptations [37]. On the other hand, we combined studies with participants of wide age ranges, including individuals aged 60 to 90 years. In this regard, aging is associated with an impaired anabolic response to both aminoacidemia and acute physical exercise [38], which might have acted as a confounding factor in our results. Interestingly, the study that included older adults was the one reporting the greatest increases in muscle strength [17]. Thus, BP supplementation might be particularly useful for this population segment. However, statistical analyses showed no heterogeneity between studies for the outcomes that we analyzed. 

On the other hand, the benefits of supplementing with BP—and of every diet in general—should be viewed under the context of its sustainability, that is, it should have a low environmental impact that contributes to food and nutrition security and to a healthy lifestyle for present and future generations [39]. Diets that primarily contain animal-derived food sources are associated with higher greenhouse gas emissions, and indeed beef production/consumption is the food source that results in greater gas emissions (>70 kg CO_2_/kg) [40]. By contrast, the production of dairy products (such as milk or WP), and especially of plant-based protein foods, is associated with lower moderate greenhouse gas emissions [41]. Thus, a balance between environmental sustainability and optimal dietary intake (particularly regarding protein consumption) should be sought, and WP might be a more recommendable option in this regard. 

## 5. Conclusions

This systematic review and meta-analysis of randomized controlled trials showed no mid/long-term (≥4 weeks) differences between BP and WP on total daily protein intake or body composition (LBM, fat mass), and thus both protein sources might be similarly effective to promote increases in LBM. However, BP resulted in a greater total daily protein intake than NP, and our results suggest that BP might represent an effective strategy for increasing LBM and lower-limb muscle strength. However, these and other potential benefits (including preliminary data on heme iron intake and ferritin levels) should be confirmed in future studies. 

## Figures and Tables

**Table 1:** Main characteristics of the included studies.

Study	Participants and Group Assignment	Duration	Exercise Training Protocol (Common to all Study Groups)	Baseline Protein Intake	BP Group	WP Group	NP Group	Additional Group	Measurements	Main Outcomes
Burke et al. [20]	28 runners (18–24 years, 14 female) stratified by iron status, use of iron supplements, and gender, and randomized into a BP (*n* = 14) or NP group (*n* = 14)	8 weeks	Maintained their typical exercise regime	~1.7 g/kg/day	255 g of lean beef supplement per week + multivitamin daily	–	Multivitamin daily	–	-Dietary intake -Blood analysis -Body composition (plethysmography) -VO_2max_	-No differences in body composition -↑heme iron intake in females -↑hematocrit in females -No differences in VO_2max_
Daly et al. [17]	100 females (60–90 years) randomized into a BP (*n* = 53) or NP group (*n* = 47)	4 months	RT 2 times per week	~1.1–1.3 g/kg/day	220 g lean red meat (45 g protein) 6 days per week + 1 vitamin D3 capsule (1000-IU) daily	–	1 serving pasta or rice daily (25–35 g CHO) + 1 vitamin D3 capsule (1000-IU) daily	–	-Dietary intake -Physical activity -Body composition (DXA) -Muscle and fat CSA and muscle density (peripheral quantitative computed tomography) -Muscle function (TUG, FSST, and 30-s STS) -Strength (1RM estimated from 3 RM) -Blood analysis	-↑Protein intake -↑LBM -↑Leg LBM -No differences in BMD -↑Muscle strength -No differences in muscle function. -↑IGF-I -↓IL-6 -No differences in blood lipids or blood pressure.
Naclerio et al. [21]	24 active males (~26–29 years) randomized into a BP, WP or NP group (*n*=8 each)	8 weeks	RT 3 times per week	~1.5 g/kg/day	20 g of beef supplement (16.4 g protein) + 250 mL of orange juice per day	20 g of WP + 250 mL of orange juice per day	20 g of CHO + 250 mL of orange juice per day	–	-Body composition (plethysmography) -Limb circumference -Strength (1 RM) -Muscle thickness (ultrasound)	-↑*biceps brachialis* thickness -No differences in limb circumference or body composition. -No differences in strength.
Naclerio et al. [22]	24 male master triathletes (35–60 years) randomized into a BP, WP or NP group (*n* = 8 each)	10 weeks	ET 4–6 times per week	~1.3–1.5 g/kg/day	20 g of beef supplement (16.4 g protein) per day	20 g of WP per day	20 g of CHO per day	–	-Body composition (plethysmography) -VO_2max_ -Muscle thickness (ultrasound) -Blood analysis	-No differences in body composition. -↓BM -↑heme iron intake. -↑ferritin concentrations -↑muscle thickness -No differences in VO_2max_
Naclerio et al. [19]	27 active males and females (~24–28 years) randomized into a BP, WP or NP group (*n* = 8 each)	8 weeks	RT 3 times per week	~1.1–1.5 g/kg/day	20 g of beef supplement (16.4 g protein) + 250 mL of orange juice per day	20 g of WP + 250 mL of orange juice per day	20 g of CHO + 250 mL of orange juice per day	–	-Body composition (plethysmography) -Strength (total weight lifted) -Muscle thickness (ultrasound) -Blood analysis -Saliva analysis	-No differences in body composition nor muscle thickness. -↓HNP1-3 and saliva flow rate. -No differences in strength.
Negro et al. [26]	26 male and female healthy subjects (~24 years) randomized into a BP (*n* = 12) or NP group (*n* = 14)	8 weeks	RT 3 times per week	~1.0 g/kg/day	135 g (20 g protein) of tinned beef per day	–	No supplement provided	–	-Strength (1 RM) -Body composition (bioimpedance)	-↑FFM and ↓FM -No differences in LBM -No differences in strength.
Sharp et al. [18]	41 male and female trained subjects (18–30 years) randomized into a WP (*n* = 10, 5 male), BP (*n* = 10, 5 male), chicken protein (*n* = 11, 5 male) or NP group (*n* = 10, 4 male)	8 weeks	RT 3 times per week and HIIT 2 times per week.	~2.0–2.2 g/kg/day	46 g of isolated BP per day	46 g of WP per day	46 g of CHO per day	46 g of chicken protein per day	-Dietary intake -Body composition (DXA). -Anaerobic peak power (10-second sprint) -Strength (1RM) -Gastrointestinal symptoms	-↑LBM and ↓FM compared to CHO. -No differences in body composition compared to other protein sources. -No differences in strength. -↓improvement in peak power compared to WP. -No differences in gastrointestinal symptoms.

Abbreviations: BM, body mass; BMD, bone mineral density; BP, beef protein; CHO, carbohydrates; CSA, cross-sectional area; DXA, dual-energy X-ray absorptiometry; ET, endurance training; FFM, fat-free mass; FM, fat mass; FSST, 4-square step test; HIIT, high intensity interval training; HNP1-3, human neutrophil peptides; IGF-1, insulin-like growth factor 1; IL-6, interleukin-6; LBM, lean body mass; NP, no protein supplementation; RM, repetition maximum; RT, resistance training; STS, sit-to-stand test; TUG, timed-up-and-go test; VO_2max_, maximal oxygen uptake; WP, whey protein. ↑, increase; ↓, reduce.

## References

[1] Cermak N.M., Res P.T., de Groot L.C., Saris W.H.M., Loon L.J.C. (2012). Van Protein supplementation augments the adaptive response of skeletal muscle to resistance-type exercise training: A meta-analysis. Am. J. Clin. Nutr..

[2] Morton R.W., Murphy K.T., McKellar S.R., Schoenfeld B.J., Henselmans M., Helms E., Aragon A.A., Devries M.C., Banfield L., Krieger J.W. (2018). A systematic review, meta-analysis and meta-regression of the effect of protein supplementation on resistance training-induced gains in muscle mass and strength in healthy adults. Br. J. Sports Med..

[3] Pasiakos S.M., McLellan T.M., Lieberman H.R. (2014). The Effects of Protein Supplements on Muscle Mass, Strength, and Aerobic and Anaerobic Power in Healthy Adults: A Systematic Review. Sports Med..

[4] Stokes T., Hector A.J., Morton R.W., McGlory C., Phillips S.M. (2018). Recent perspectives regarding the role of dietary protein for the promotion of muscle hypertrophy with resistance exercise training. Nutrients.

[5] Elliot T.A., Cree M.G., Sanford A.P., Wolfe R.R., Tipton K.D. (2006). Milk ingestion stimulates net muscle protein synthesis following resistance exercise. Med. Sci. Sports Exerc..

[6] Moore D., Robinson M., Fry J., Tang J., Glover E., Wilkinson S., Prior T., Tarnopolsky M., Philips S. (2009). Ingested protein dose response of muscle and albumin protein synthesis after resistance exercise in young men. Am. J. Clin. Nutr..

[7] Yang Y., Churchward-Venne T.A., Burd N.A., Breen L., Tarnopolsky M.A., Phillips S.M. (2012). Myofibrillar protein synthesis following ingestion of soy protein isolate at rest and after resistance exercise in elderly men. Nutr. Metab..

[8] Joy J.M., Lowery R.P., Wilson J.M., Purpura M., De Souza E.O., Wilson S.M., Kalman D.S., Dudeck J.E., Jäger R. (2013). The effects of 8 weeks of whey or rice protein supplementation on body composition and exercise performance. Nutr. J..

[9] Duff W., Chilibeck P., Rooke J., Kaviani M., Krentz J., Haines D. (2014). The Effect of Bovine Colostrum Supplementation in Older Adults during Resistance Training. Int. J. Sport Nutr. Exerc. Metab..

[10] Cribb P.J., Williams A.D., Hayes A., Carey M.F. (2006). The Effect of Whey Isolate and Resistance Training on Strength, Body Composition and Plasma Glutamine. Int. J. Sport Nutr. Exerc. Metab..

[11] Volek J.S., Volk B.M., Gómez A.L., Kunces L.J., Kupchak B.R., Freidenreich D.J., Aristizabal J.C., Saenz C., Dunn-Lewis C., Ballard K.D. (2013). Whey Protein Supplementation During Resistance Training Augments Lean Body Mass. J. Am. Coll. Nutr..

[12] Gorissen S.H.M., Rémond D., van Loon L.J.C. (2015). The muscle protein synthetic response to food ingestion. Meat Sci..

[13] Symons T., Schutzler S.E., Cocke T.L., Chinkes D.L., Wolfe R.R., Paddon-Jones D. (2007). Aging does not impair the anabolic response to a protein-rich meal. Am. J. Clin. Nutr..

[14] Symons T., Sheffield-Moore M., Wolfe R.R., Paddon-Jones D. (2009). A Moderate Serving of High-Quality Protein Maximally Stimulates Skeletal Muscle Protein Synthesis in Young and Elderly Subjects. J. Am. Diet. Assoc..

[15] Robinson M.J., Burd N.A., Breen L., Rerecich T., Yang Y., Hector A.J., Baker S.K., Phillips S.M. (2013). Dose-dependent responses of myofibrillar protein synthesis with beef ingestion are enhanced with resistance exercise in middle-aged men. Appl. Physiol. Nutr. Metab..

[16] Symons T., Sheffield-Moore M., Mamerow M.M., Wolfe R.R., Paddon-Jones D. (2011). The anabolic response to resistance exercise and a protein-rich meal is not diminished by age. J. Nutr. Health Aging.

[17] Daly R.M., O’Connell S.L., Mundell N.L., Grimes C.A., Dunstan D.W., Nowson C.A. (2014). Protein-enriched diet, with the use of lean red meat, combined with progressive resistance training enhances lean tissue mass and muscle strength and reduces circulating IL-6 concentrations in elderly women: A cluster randomized controlled trial. Am. J. Clin. Nutr..

[18] Sharp M.H., Lowery R.P., Shields K.A., Lane J.R., Gray J.L., Partl J.M., Hayes D.W., Wilson G.J., Hollmer C.A., Minivich J.R. (2018). The Effects of Beef, Chicken, or Whey Protein Post-Workout on Body Composition and Muscle Performance. J. Strength Cond. Res..

[19] Naclerio F., Larumbe-Zabala E., Ashrafi N., Seijo M., Nielsen B., Allgrove J., Earnest C.P. (2017). Effects of protein–carbohydrate supplementation on immunity and resistance training outcomes: A double-blind, randomized, controlled clinical trial. Eur. J. Appl. Physiol..

[20] Burke D.E., Johnson J.V., Vukovich M.D., Kattelmann K.K. (2012). Effects of Lean Beef Supplementation on Iron Status, Body Composition and Performance of Collegiate Distance Runners. Food Nutr. Sci..

[21] Naclerio F., Seijo-Bujia M., Larumbe-Zabala E., Earnes C. (2017). Carbohydrates Alone or Mixing With Beef or Whey Protein Promote Similar Training Outcomes in Resistance Training Males: A Double Blind, Randomized Controlled Clinical Trial. Int. J. Sport Nutr. Exerc. Metab..

[22] Naclerio F., Seijo M., Larumbe-Zabala E., Ashrafi N., Christides T., Karsten B., Nielsen B.V. (2017). Effects of Supplementation with Beef or Whey Protein Versus Carbohydrate in Master Triathletes. J. Am. Coll. Nutr..

[23] Moher D., Liberati A., Tetzlaff J., Altman D.G., Altman D., Antes G., Atkins D., Barbour V., Barrowman N., Berlin J.A. (2009). Preferred reporting items for systematic reviews and meta-analyses: The PRISMA statement. PLoS Med..

[24] Higgins J.P.T., Altman D.G., Gøtzsche P.C., Jüni P., Moher D., Oxman A.D., Savović J., Schulz K.F., Weeks L., Sterne J.A.C. (2011). The Cochrane Collaboration’s tool for assessing risk of bias in randomised trials. BMJ.

[25] Rosenthal R. (1991). Meta-Analytic Procedures for Social Research.

[26] Negro M., Vandoni M., Ottobrini S., Codrons E., Correale L., Buonocore D., Marzatico F. (2014). Protein supplementation with low fat meat after resistance training: Effects on body composition and strength. Nutrients.

[27] Van Vliet S., Burd N.A., van Loon L.J.C. (2015). The Skeletal Muscle Anabolic Response to Plant- versus Animal-Based Protein Consumption. J. Nutr..

[28] Tang J.E., Moore D.R., Kujbida G.W., Tarnopolsky M.A., Phillips S.M. (2009). Ingestion of whey hydrolysate, casein, or soy protein isolate: Effects on mixed muscle protein synthesis at rest and following resistance exercise in young men. J. Appl. Physiol..

[29] Burd N.A., Yang Y., Moore D.R., Tang J.E., Tarnopolsky M.A., Phillips S.M. (2012). Greater stimulation of myofibrillar protein synthesis with ingestion of whey protein isolate v. micellar casein at rest and after resistance exercise in elderly men. Br. J. Nutr..

[30] Burd N.A., Gorissen S.H., Van Vliet S., Snijders T., Van Loon L.J.C. (2015). Differences in postprandial protein handling after beef compared with milk ingestion during postexercise recovery: A randomized controlled trial. Am. J. Clin. Nutr..

[31] Trumbo P., Schlicker S., Yates A., Poos M. (2002). Dietary reference intakes for energy, carbohydrate, fiber, fat, fatty acids, cholesterol, protein and amino acids. J. Am. Diet. Assoc..

[32] Wu G. (2016). Dietary protein intake and human health. Food Funct..

[33] Traylor D.A., Gorissen S.H.M., Phillips S.M. (2018). Perspective: Protein requirements and optimal intakes in aging: Arewe ready to recommend more than the recommended daily allowance?. Adv. Nutr..

[34] Trommelen J., Betz M.W., van Loon L.J.C. (2019). The Muscle Protein Synthetic Response to Meal Ingestion Following Resistance-Type Exercise. Sports Med..

[35] Pennings B., Groen B.B.L., Van Dijk J.W., De Lange A., Kiskini A., Kuklinski M., Senden J.M.G., Van Loon L.J.C. (2013). Minced beef is more rapidly digested and absorbed than beef steak, resulting in greater postprandial protein retention in older men. Am. J. Clin. Nutr..

[36] Egan B., Zierath J.R. (2013). Exercise metabolism and the molecular regulation of skeletal muscle adaptation. Cell Metab..

[37] Moore D.R., Camera D.M., Areta J.L., Hawley J. (2014). A Beyond muscle hypertrophy: Why dietary protein is important for endurance athletes. Appl. Physiol. Nutr. Metab..

[38] Breen L., Phillips S.M. (2011). Skeletal muscle protein metabolism in the elderly: Interventions to counteract the “anabolic resistance” of ageing. Nutr. Metab..

[39] MacDiarmid J.I. (2013). Is a healthy diet an environmentally sustainable diet?. Proc. Nutr. Soc..

[40] Scarborough P., Appleby P.N., Mizdrak A., Briggs A.D.M., Travis R.C., Bradbury K.E., Key T.J. (2014). Dietary greenhouse gas emissions of meat-eaters, fish-eaters, vegetarians and vegans in the UK. Clim. Chang..

[41] Gorissen S.H.M., Witard O.C. (2018). Characterising the muscle anabolic potential of dairy, meat and plant-based protein sources in older adults. Proc. Nutr. Soc..

